# Fe^2+^-Sensing α-Synuclein Iron-Responsive Messenger RNA/eIF4F Complex Binding and Regulating mRNA Translation Activation and Repression

**DOI:** 10.3390/ijms26199320

**Published:** 2025-09-24

**Authors:** Mateen A. Khan

**Affiliations:** Department of Life Sciences, College of Science & General Studies, Alfaisal University, Riyadh 11533, Saudi Arabia; matkhan@alfaisal.edu

**Keywords:** Parkinson’s disease, α-Syn IRE, eIF4F, IRP1, fluorescence, binding, thermodynamics, protein synthesis

## Abstract

Alpha-synuclein (α-Syn) protein plays a crucial role in the pathophysiology of Parkinson’s disease (PD). In the 5′-untranslated regions (5′-UTRs) of α-Syn, mRNA has a structured iron-responsive element (IRE) with a stem loop that regulates translation. Iron (labile as Fe^2+^) enhances protein synthesis rates through an IRE mRNA. This investigation aimed to describe the way in which α-Syn IRE interacts with eIF4F and establish a relationship between binding affinity and translation efficiency. The strong binding affinity of α-Syn IRE with eIF4F was demonstrated by a fluorescence-based experiment, with *K*_a_ = 8.4 × 10^6^ M^−1^ at 25 °C. Fe^2+^ further increased (~three-fold) the affinity of α-Syn IRE with eIF4F, outcompeting binding with IRP1. With an increase in temperature (10–30 °C), *K*_d_ values increased from 35.8 ± 1.6 nM to 158 ± 8.7 nM for the interaction of α-Syn IRE with eIF4F; however, adding Fe^2+^ demonstrated significantly increased affinity throughout the same temperature range. Thermodynamic analyses demonstrated that α-Syn IRE/eIF4F binding occurred spontaneously, with the presence of van der Waals and hydrogen bonding. Fe^2+^ enhanced the α-Syn IRE/eIF4F complex’s change in enthalpic and binding free energy contributions, which led to a more stable complex formation through the involvement of more hydrogen bonding. Exogenous addition of eIF4F in depleted WG or RR lysates restored α-Syn protein synthesis. Fe^2+^ further boosted α-Syn mRNA translation. IRP1 repressed α-Syn translation, although the addition of Fe^2+^ reversed this effect by boosting activator eIF4F binding and decreasing repressor IRP1 binding. These findings reveal the significance of iron in the α-synuclein mRNA regulatory process and validate its contribution as a strong enhancer of α-Syn mRNA translation.

## 1. Introduction

Parkinson’s disease (PD), the second most common neurodegenerative illness, is characterized by bradykinesia, stiffness, postural instability, and tremors [1]. The existence of α-synuclein (α-Syn) aggregates in Lewy bodies is the primary pathogenesis of Parkinson’s disease (PD), while the exact cause is still unknown [2]. Autosomal dominant PD is brought on by mutations in the *SNCA* gene [3]. A heritable variant of the illness is also brought on by the usual gene being duplicated or tripled. Thus, based on early research, α-Syn plays critical role in the pathogenesis of PD and other α-synucleinopathies [4].

α-Syn is mostly found in the brain and is inherently disordered. Through oligomerization, misfolding, and fibril formation, this protein can spread throughout brain neurons and build up in Lewy bodies and Lewy neurites [5]. These conditions cause dementia and neuronal death [6]. Given that people with *SNCA* gene locus multiplication acquire dominantly inherited PD and dementia with a gene dosage effect, the level of α-Syn expression is a significant determinant of fibrillization rate and neurotoxicity [7]. Metal ion dysregulation, lysosomal malfunction, and mitochondrial impairment are the mechanisms by which abnormal versions of α-Syn cause selective and gradual neuronal death. Because it interacts with proteins involved in neurotransmitter release and reuptake, α-Syn is primarily found at presynaptic locations [8]. PD may develop as a result of a variety of factors, including genetics, aging, and environmental exposures. Further highlighting the intricacy of neurodegenerative processes, epigenetic changes, such as histone deacetylation, have also been linked to neurological disorders [9,10]. A number of post-translational changes, including phosphorylation, can impact α-Syn aggregation and folding, which is crucial in Parkinson’s disease. Because of this, the variables controlling the spread of pathogenic α-Syn forms are still up for question about the vulnerability functions in the context of spread.

It has been demonstrated in multiple investigations that α-Syn overexpression results in Fe-dependent aggregation [11]. PD has been linked to α-Syn dysfunction since it is more prevalent in Lewy bodies along with iron. α-Syn oligomerization may play a role in the oxidative damage that iron causes to the brain during Parkinson’s disease pathogenesis, while disturbance of iron homeostasis can result in neurotoxicity through several pathways including in common neurodegenerative illnesses like Parkinson’s disease. Brain Fe deposit enhances with age, which varies by anatomical location. It has been reported Fe accumulation is enhanced in substantia nigra, neurofibrillary tangles, and Lewy bodies of PD patients [12,13]. An increased risk of PD pathology is also associated with an enhanced amount of Fe with brain aging [14]. A recently identified iron-responsive element (IRE) stem–loop structure in the 5′-noncoding region of the *α-Syn* gene may be a possible PD therapy target by blocking the translation of the gene that causes PD.

RNA–protein interactions influence α-Syn mRNA splicing, which is essential for many basic biological processes [15]. Interaction of regulatory protein can change the differential translation of a single messenger RNA through selection of a particular translation initiation codon in addition to alternative splicing [16,17,18]. Through the 5′-noncoding region, sequence-specific RNA-binding proteins can effectively suppress translation in mammals [19]. The 3′-UTR is another crucial regulatory element. All mRNAs have this poly(A) tail, which, depending on its length and the binding of specific regulatory proteins, can either upregulate or downregulate translation [16]. An iron-responsive element, whose role in Fe homeostasis has already been documented, is the most well-studied example of a short structural element inside the 5′-UTR that influences the translation of eukaryotic mRNAs. Since the preinitiation ribosomal complex interacts at the site, the stem–loop IRE is often found within fifty nucleotides of 5′-mRNA sites. This distance is crucial for its functionality. Iron-regulatory proteins (IRPs) bind to IRE and regulate translation. Recently, a functional stem–loop IRE linked to the expression of α-Syn and an amyloid precursor protein (APP) have been reported [20,21]. It was reported that this IRE conferred iron-dependent regulation and was similar to those identified in the 5′-noncoding regions of the mammalian TfR and ferritin RNAs. The 5′-UTR of α-Syn mRNA contains a structured IRE that controls translation. By a mechanism akin to ferritin mRNA, iron was shown to regulate the translation of α-Syn [22]. At low iron concentrations, IRP binds to stem–loop IRE. At high concentrations, IRP frees the mRNA to undergo translation [23,24]. Translational regulation of α-Syn via Fe^2+^, IRE, and Fe in Lewy bodies supports their combined roles in PD [25,26].

Translation starts when eukaryotic initiation factor (eIF) 4F binds with the mRNA cap structure and ribosome. IRP may also be contacted by the supramolecular complex known as eIF4F, which is made up of eIF4G (scaffolding protein), eIF4B (RNA binding protein), and eIF4A (helicase) [27]. Ribosome binding is a complex process since it requires the binding of numerous protein components to assemble mRNA, initiator tRNA, and both ribosome subunits together. Due to its competitive binding with IRP1 to IRE, which indicates that eIF4F and IRP binds to the overlapping or same sites, eIF4F binding can promote a quicker response to cellular iron levels [21,28]. Two regulatory proteins, eIF4F (activator) and IRP (repressor), have been shown to be competitively bound to IRE [28,29]. Fe^2+^ activates eIF4F binding and inhibits IRP1 binding to IRE [29] and activates eIF4F binding [30].

The aim of present study was to comprehend the interaction of α-Syn IRE with eIF4F and correlate binding with in vitro translation efficiency. Fe^2+^ increased α-Syn IRE’s affinity for initiation factor 4F whilst lowering its affinity for IRP1 [31]. Two proteins, eIF4F and IRP1, interact competitively with α-Syn IRE. Addition of Fe^2+^ restored α-Syn mRNA translation when exogenous eIF4F was supplemented in depleted wheat germ lysate or rabbit reticulocyte lysate. These results indicate that the Fe^2+^ level plays a critical role in regulating α-synuclein expression in PD.

## 2. Results

### 2.1. α-Syn IRE Binds Strongly to eIF4F

**Figure 1:**
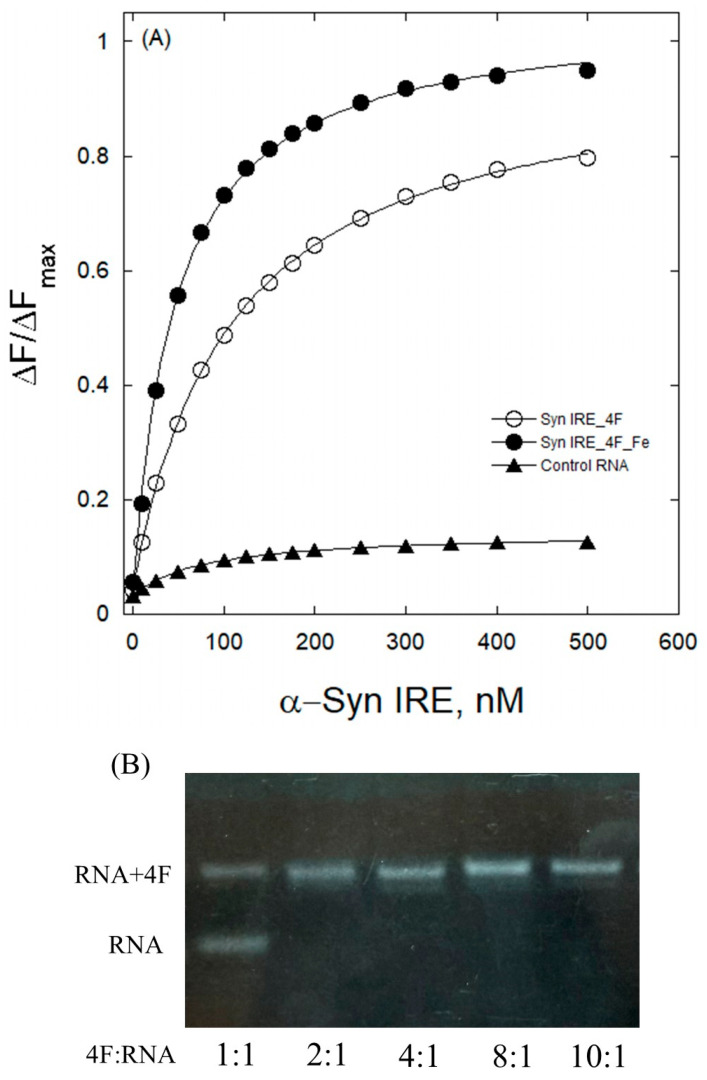
Interaction of α-Syn IRE RNA with eIF4F. (**A**) Binding affinity of α-Syn IRE with eIF4F in the absence and presence of Fe^2+^. ΔF/ΔF_max_ values for eIF4F (○) and eIF4F_Fe (●) versus Syn IRE concentration are shown. As explained in the Materials and Methods section, the curves were fitted to obtain *K*_d_. Concentrations were as follows: eIF4F, 50 nM; Syn IRE, 0–500 nM; and Fe^2+^, 50 μM. Samples were prepared by incubation of Syn IRE, initiation factor, and Fe^2+^ for 15 min at 25 °C. An oligonucleotide (▲, 30-nt 5S RNA, used as a negative control) did not bind to eIF4F. (**B**) α-Syn IRE RNA binding to eIF4F was measured by an electrophoretic mobility shift assay. A constant IRE RNA concentration (0.1 μM) was used with varying concentrations of eIF4F protein (0.1–1.0 μM). IRE RNA was stained with ethidium bromide.

Fluorescence emission quenching experiments were conducted with and without Fe^2+^ to examine α-Syn IRE’s interaction with translation initiation factor (eIF) 4F. A decrease in fluorescence of eIF4F protein with the addition of α-Syn IRE can be used to characterize binding when α-Syn IRE and eIF4F protein form an association complex. By adding α-Syn IRE at variable concentrations (0–0.5 μM) at 298 K, the fluorescence of eIF4F decreased in a concentration-dependent manner, revealing α-Syn IRE/eIF4F binding (Figure 1A). Adding α-Syn IRE to eIF4F changes the intensity of eIF4F, which reflects the interaction between Syn RNA and eIF4F protein. Following protein λ_ex_ (excitation wavelength) at 280 nM, the spectra of native protein (eIF4F) fluorescence had a maximum peak, λ_em_ (emission wavelength), at 334 nm. Conversely, there was a noticeable drop in eIF4F fluorescence intensity with adding variable concentrations of α-Syn IRE, demonstrating the formation of an association complex between α-Syn IRE and eIF4F.

Figure 1A shows the fluorescence-based binding curve for the interaction of α-Syn IRE with eIF4F in the absence and presence of Fe^2+^. The level of protein fluorescence quenching was shown to be associated with the degree of α-Syn IRE bound with eIF4F, which further suggests association of the RNA/protein complex. A change in protein fluorescence occurred at 334 nm, suggesting the presence of aromatic chromophores, tryptophan, phenylalanine, and tyrosine, which are concealed by the folding of eIF4F protein due to Syn IRE interaction. This interaction brings aromatic chromophores closer. This folding of protein caused an alteration in the intensity of the spectrum through changing the electronic distribution of fluorophores [32]. α-Syn IRE strongly interacts eIF4F at a dissociation constant value in the nanomolar scale (*K*_d_ = 119.2 ± 4.4 nM), which indicates a relatively weaker affinity as compared to the binding affinity for ferritin [33] and APP IRE [21] at 298 K. However, under similar experimental conditions, eIF4F did not show binding to the 30-oligoribonucleotide stem loop 5S RNA, suggesting that eIF4F protein specifically interacts with α-Syn IRE, as reported earlier for APP and ferritin IRE [28,33]. eIF4F’s strong interaction with α-Syn IRE, even in the absence of the mRNA cap, explains the previous findings that protein synthesis is significantly affected by removing the IRE mRNA stem–loop structure but not by removing the IRE mRNA cap [34].

For the titration, we employed a constant eIF4F protein concentration (0.1 μM) in order to analyze the Syn IRE/eIF4F interactions using fluorescence quenching. To collect data using the gel-electrophoretic mobility shift test, similar to previous investigations of IRE/protein complexes, and to assess the potential impact of titrating with varying protein concentrations and employing a constant Syn IRE concentration [35,36], we used the electrophoretic mobility shift test to investigate the Syn IRE/eIF4F interactions. To obtain an eIF4F/Syn IRE molar ratio between 1.0 and 10, a constant Syn IRE concentration (0.1 μM) was combined with a variable eIF4F initiation factor protein concentration (0.1–1.0 μM). The easiest way to see the differences between the Syn IRE and eIF4F interaction in the gels is to compare the unshifted Syn IRE RNA bands (Figure 1B) compared to the Syn IRE/eIF4F complex. The stability of the Syn IRE/eIF4F complex bands in the gels allowed for the direct observation of RNA and protein interactions. There was no change in the reaction mixture when the Syn IRE and eIF4F proteins were added in a different order.

### 2.2. Fe^2+^ Affect Interaction of α-Syn IRE with eIF4F

The complex binding affinity was further evaluated to see if there were any notable variations between the interaction of α-Syn IRE with eIF4F after adding Fe^2+^. A further reduction in the α-Syn IRE/eIF4F association complex intensity was observed upon the addition of Fe^2+^. After adding 50 μM Fe^2+^, the samples were kept in anaerobic (–O_2_) conditions to further examine the α-Syn IRE binding affinity for eIF4F. By adding Fe^2+^, the eIF4F spectral signal at λ_em_ = 334 nm and λ_ex_ = 280 nm reduced even further in the titration experiment with different α-Syn IRE concentrations. The fluorescence intensity curves for the association of α-Syn IRE/eIF4F with and without Fe^2+^ differ significantly, as can be seen in Figure 1A. The fluorescence-based binding curve for the α-Syn IRE/eIF4F complex was considerably reduced by Fe^2+^. Fe^2+^ induced structural changes to the α-Syn IRE/eIF4F complex, which resulted in the creation of a more stable complex. The binding affinity of α-Syn IRE for eIF4F at 298 K was boosted (~three-fold) by the addition of Fe^2+^ (*K*_d_ = 43.7 ± 2.7 nM for α-Syn IRE-Fe^2+^/eIF4F; *K*_d_ = 119.2 ± 4.4 nM for α-Syn IRE/eIF4F) (Table 1). The specificity of the Fe^2+^-influenced enhanced binding of α-Syn IRE with eIF4F is quantifiably supported by the differences in the complex’s binding affinity in the presence of Fe^2+^. It has previously been demonstrated that Fe^2+^ stabilizes APP [21] and ferritin IRE/eIF4F [28] complex and Fe^2+^ removes IRE RNA from the α-Syn [31], ferritin [35], and amyloid precursor protein [29] RNA complex with IRP1, suggesting a regulatory role of iron for the binding of the two proteins for mRNA.

### 2.3. Temperature Effects on α-Syn IRE/eIF4F Binding

**Figure 2:**
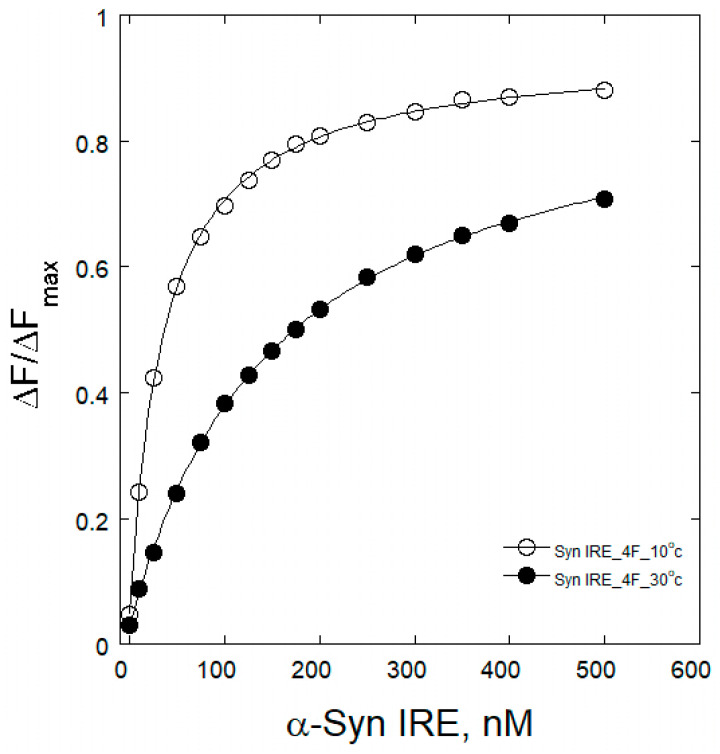
Binding plots between α-Syn IRE and eIF4F at variable temperatures. *K*_d_ was obtained by fitting non-linear curves. Figure 1 legends are explained in the experimental conditions.

**Figure 3:**
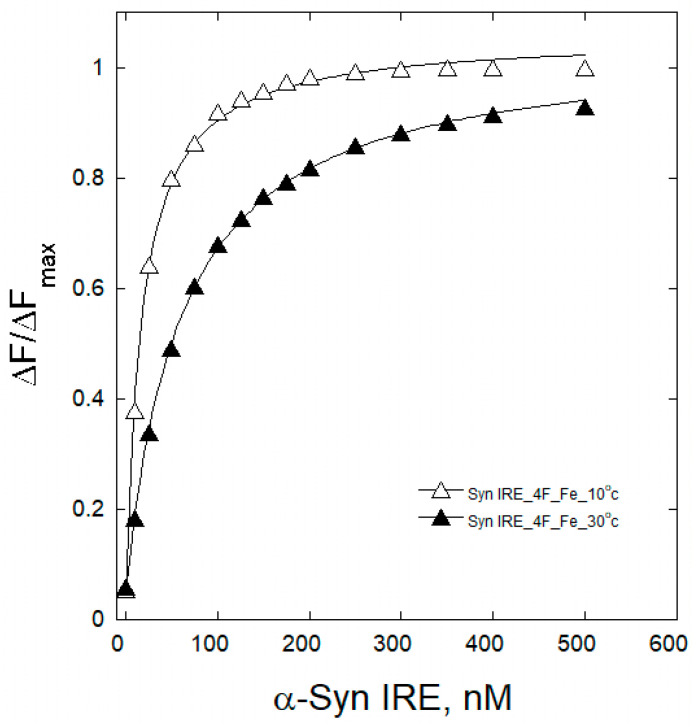
Fe^2+^-dependent binding plots for α-Syn IRE and eIF4F at variable temperatures. Fe^2+^ concentration was 50 μM.

**Figure 4:**
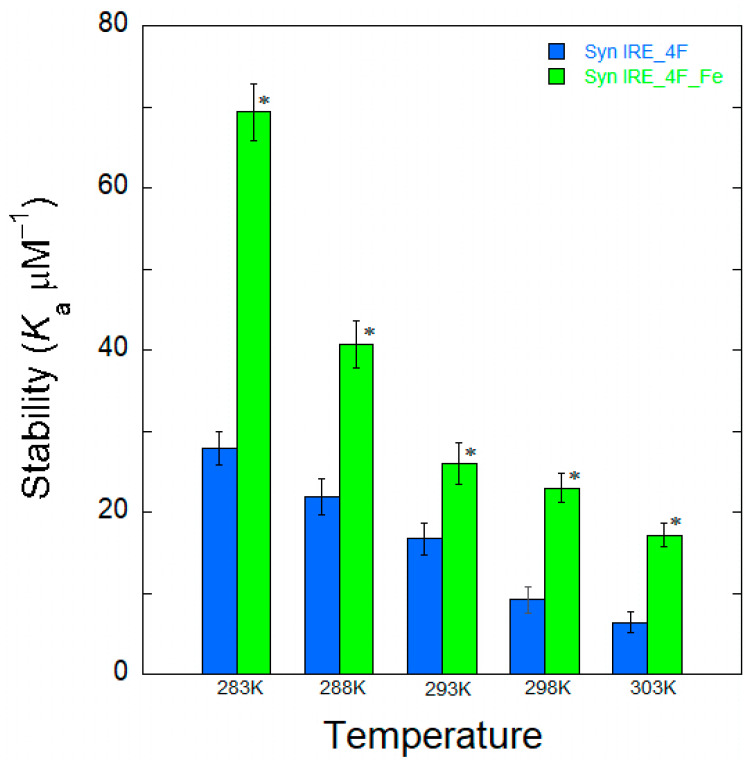
Histogram representation of the temperature-dependent affinity constant for α-Syn IRE with eIF4F with or without Fe^2+^ (50 μM). The values shown are the average of three separate experiments. * *p* < 0.01, as determined by Student’s two-tailed *t*-test; results were significantly different with added Fe^2+^. The values plotted are means ± S.E. of Syn IRE–eIF4F binding with added iron.

To understand the interaction mechanism between Syn IRE and eIF4F, the change in binding association at varying temperatures was observed. An internal understanding of the quenching process at different temperatures for the binding of α-Syn IRE to eIF4F interaction provides a comprehensive mechanism. Variations in temperature were therefore taken into account for the fluorescence binding assays. Temperature-dependent fluorescence emission tests were conducted at five different temperatures (283, 288, 293, 298, and 303 K) for the interaction of α-Syn IRE and eIF4F, either with or without Fe^2+^. Upon adding α-Syn IRE, temperature-dependent reductions in eIF4F fluorescence intensity were noted. A drop in the sample’s fluorescence intensity between 283 K and 303 K provided information about the impact of temperature on the formation of the eIF4F and Syn RNA association. Temperature-dependent fluorescence plots of α-Syn IRE’s interaction with eIF4F at 283 K and 303 K in the absence or presence of Fe^2+^ are shown in Figure 2 and Figure 3. Calculated values of temperature-dependent binding constant data are shown in Table 1 and Figure 4. The dissociation constant (*K*_d_) rose with temperature. This kind of temperature-dependent fluctuating binding constant points to the development of a complex between eIF4F and α-Syn IRE. The dynamic binding of the α-Syn IRE/eIF4F interface is indicated by the increase in the binding constant with temperature. The dissociation constant for the association of α-Syn IRE with eIF4F at all temperatures examined was at the nanomolar scale, according to the binding data, demonstrating a robust interaction between α-Syn IRE and eIF4F. The fluorescence quenching experiments at five different temperatures revealed that *K*_d_ increased with temperature, rising from 283 K (*K*_d_ = 35.8 ± 1.6 nM) to 303 K (*K*_d_ = 158 ± 8.7 nM). Evaluating the fluorescence experimental data revealed that α-Syn IRE interacts with eIF4F with a stronger affinity at lower temperatures compared to higher temperatures. Lower affinity values were clearly observed when the temperature increased, and this type of temperature-dependent shift in binding data implies that eIF4F and α-Syn IRE form a static complex. This result demonstrated that the α-Syn IRE/eIF4F complex was more stable at lower temperatures.

The addition of Fe^2+^ reduced the dissociation constant (*K*_d_) for α-Syn IRE’s interaction with eIF4F at all temperatures examined. At 298 K, the binding constant of α-Syn IRE with eIF4F was substantially tripled with adding Fe^2+^. The addition of Fe^2+^ increased the binding association of α-Syn IRE/eIF4F at every temperature that was examined, following the same trend as when Fe^2+^ was absent. The results showed that binding with Fe^2+^ consistently had a greater affinity for α-Syn IRE/eIF4F than binding without Fe^2+^ at all temperatures examined (Figure 4). The value of *K*_d_ clearly increased with temperature for this activity, indicating a dynamic mode of interaction.

### 2.4. Thermodynamic Analyses of eIF4F/α-Syn IRE Association

**Figure 5:**
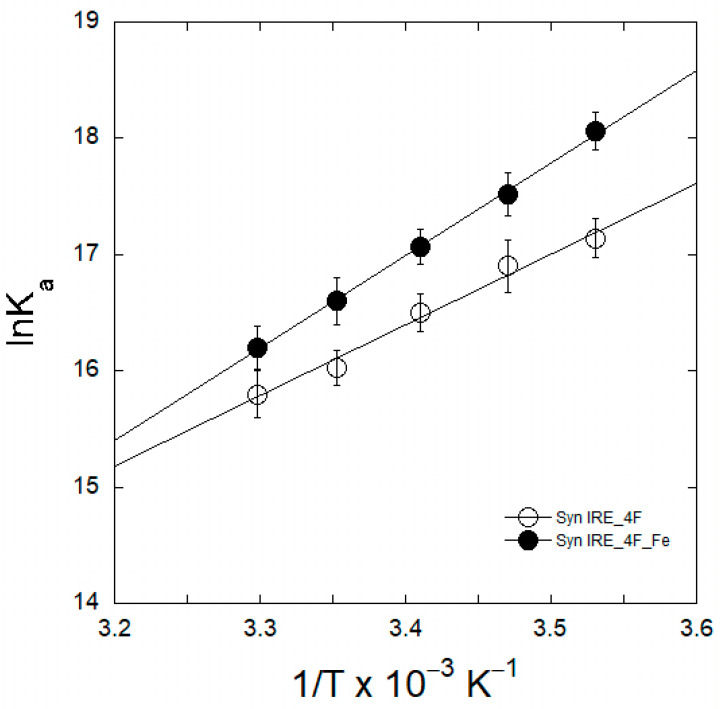
The van’t Hoff plot of ln *K*_a_ vs. temperature (T) for the association of α-Syn IRE with eIF4F.

The role of thermodynamics in the association of eIF4F/α-Syn IRE binding was assessed by tracking temperature-dependent fluorescence quenching to evaluate the specific forces involved in the stability of the functional complex between α-Syn IRE and eIF4F. The thermodynamic characteristics of the eIF4F/α-Syn IRE association required estimating the temperature-dependent binding affinity (*K*_a_ = 1/*K*_d_) data, both with and without Fe^2+^. The experimental data fits were linearly regressed using a van’t Hoff plot showing temperature versus ln *K*_a_ (Figure 5). The van’t Hoff plot slope can be linearly analyzed to yield –ΔH/R, while the intercept yields ΔS/R. Following this, the enthalpy (ΔH) and entropy (TΔS) were computed using the linear regression equations that were produced. The strong interaction between α-Syn IRE and eIF4F is unambiguously connected with a high enthalpy of association, at ΔH = −45.6 ± 2.9 kJ mol^−1^, and a negative entropy change (entropy opposed), at ΔS = −35.7 ± 3.4 J mol^−1^ K^−1^. The addition of Fe^2+^ dramatically altered the enthalpy of association and entropy, with large ΔH (−69.2 ± 3.5 kJ mol^−1^) and ΔS (−83.9 ± 4.7 J mol^−1^ K^−1^) values obtained. Stabilization of the complex formation is attributed to hydrogen bonding and van der Waals interactions, as indicated by the negative values for both ΔH and ΔS [37,38].

Using the information from Table 2 and Equation (2), the ΔG value was calculated at 298 K. The change in the binding free energy was ΔG = −33.2 ± 2.7 kJ mol^−1^ with respect to eIF4F/α-Syn IRE and ΔG = −51.9 ± 2.8 kJ mol^−1^ with respect to eIF4F/α-Syn IRE-Fe^2+^. The more negative ΔG value for α-Syn IRE/eIF4F with the addition of Fe^2+^ indicates an additional hydrogen bond for the association between α-Syn IRE and eIF4F. One possible explanation for this could be that iron creates a more advantageous molecular connection with greater affinity. Environmental and structural factors can explain the differences in ΔG values between the interactions in the presence and absence of Fe^2+^. The association of α-Syn IRE with eIF4F is impacted by Fe^2+^, which provides a different affinity, leading to a more negative ΔG value. These findings are important because they provide soft tuning of the eIF4F protein structure so that it can bind to Syn IRE more effectively without losing the molecular interactions required for the functionality of the protein.

### 2.5. Competition of eIF4F and IRP1 for α-Syn IRE Binding

**Figure 6:**
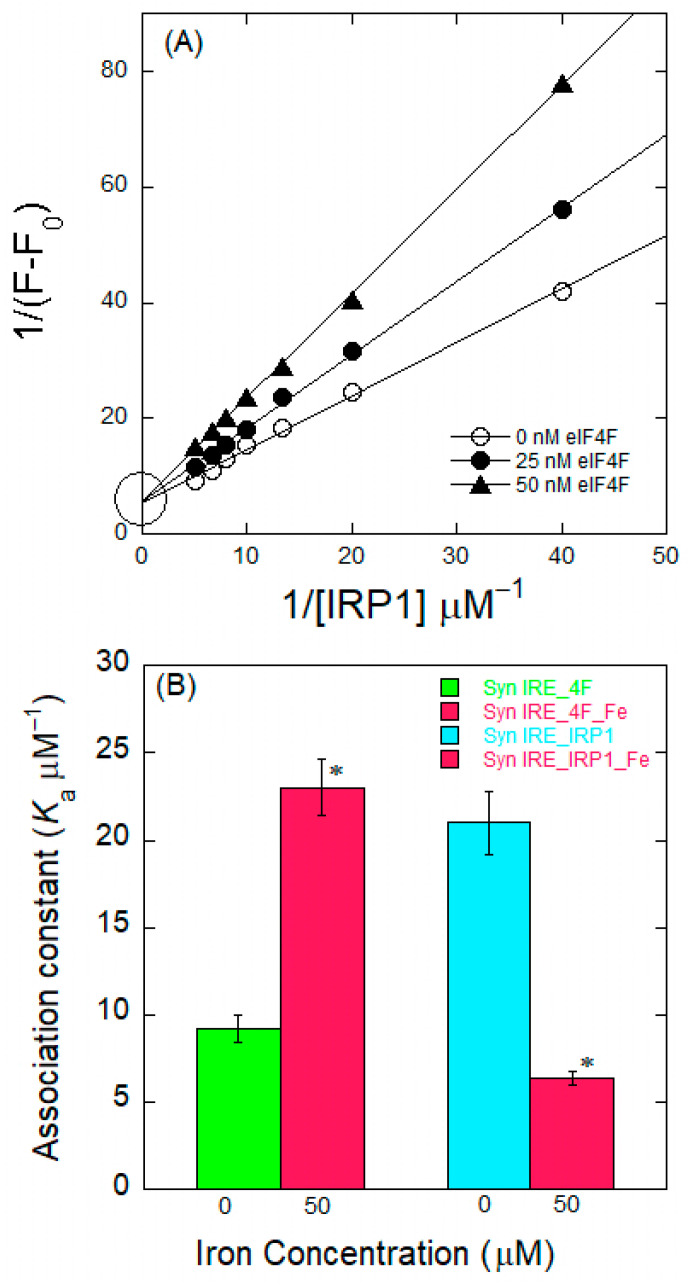
Competitive binding between IRP1 and eIF4F for α-Syn IRE. (**A**) Plot of 1/ΔF vs. 1/[IRP1] for competitive binding. Fluorescence titration plots for the competitive binding were obtained with Syn IRE as a function of IRP1 at three different concentrations (0, 25, and 50 nM) of eIF4F. Concentration of Syn IRE = 50 nM. (**B**) Fe^2+^ effects are opposite for Syn IRE’s association with IRP1 and eIF4F. Binding affinity values of Syn IRE-eIF4F ± Fe^2+^ were obtained from Figure 1, while those of Syn IRE-IRP1 ± Fe^2+^ were obtained from [31]. * *p* < 0.01, as determined by Student’s two-tailed *t*-test. The values plotted are means ± S.E. of Syn IRE-eIF4F binding with added Fe^2+^.

In order to determine whether the two proteins, eIF4F (an activator) and IRP1 (a repressor), bind to α-Syn IRE competitively or non-competitively, fluorescence changes in the 5′-fluorescein (FI)-tagged α-Syn IRE signal were assessed by titrating different concentrations of IRP1 with or without adding eIF4F. The fluorescence titrations at varying molar ratios of the Syn IRE:eIF4F (1:0, 1:0.5, and 1:1) interaction with titration with different IRP1 concentrations are shown in Figure 6A. The fluorescence intensity of ^FI^α-Syn IRE was measured in relation to the IRP1 concentration with or without adding eIF4F. A Lineweaver–Burk plot was plotted using the titration results. The competition of the two proteins’ (eIF4F, IRP1) association with α-Syn IRE is indicated by the convergent lines around similar positions on the y-axis of the plots. IRP1 and eIF4F efficiently compete for α-Syn IRE binding (Figure 6A). Convergence of the lines [1/(IRP1) vs. 1/ΔF] on the y-axis indicates competitive binding. Since the competitive inhibitor causes the substrate *K*_d_ to appear to increase by a factor of (1 + i/*K*_i_), where i is the inhibitor’s concentration and *K*_i_ is its dissociation equilibrium constant, the apparent *K*_d_ value for the α-Syn IRE/IRP1 interaction is equal to *K*_d,IRP_ (1 + [eIF4F]/*K*_d,4F_) for the competitive binding of eIF4F and IRP1. For α-Syn IRE/IRP1, *K*_d_ increases at a ratio of approximately 3:1 for Fe^2+^ (0 to 50 μM) in the absence of eIF4F (Figure 6B). Adding eIF4F gives the following: *K*_d,IRP_/*K*′_d,IRP_ = {*K*_d,IRP_ *K*′_d,4F_ (*K*_d,4F_ + [eIF4F])}/{*K*′_d,IRP_ *K*_d,4F_(*K*′_d,4F_ + [eIF4F])}, where the superscripts specify Fe^2+^ (50 μM), and the *K*_d_ subscripts specify the species. The dissociation of α-Syn IRE/IRP1 is increased by the ratio of *K*′_d_,_4F_/*K*_d_,_4F_, assuming that [eIF4F] is more related to *K*_d_,_4F_ (*K*_d_~50 nM). Consequently, when the Fe^2+^ levels become 50 μM with the addition of eIF4F, the *K*_d_ value of α-Syn IRE/IRP1 increases to 100:1 compared to α-Syn IRE/IRP1 at zero Fe^2+^ levels. Figure 6B illustrates the binding advantage of α-Syn IRE with eIF4F over IRP1 with adding Fe^2+^.

### 2.6. Effects of Fe^2+^ on α-Syn mRNA Translation

**Figure 7:**
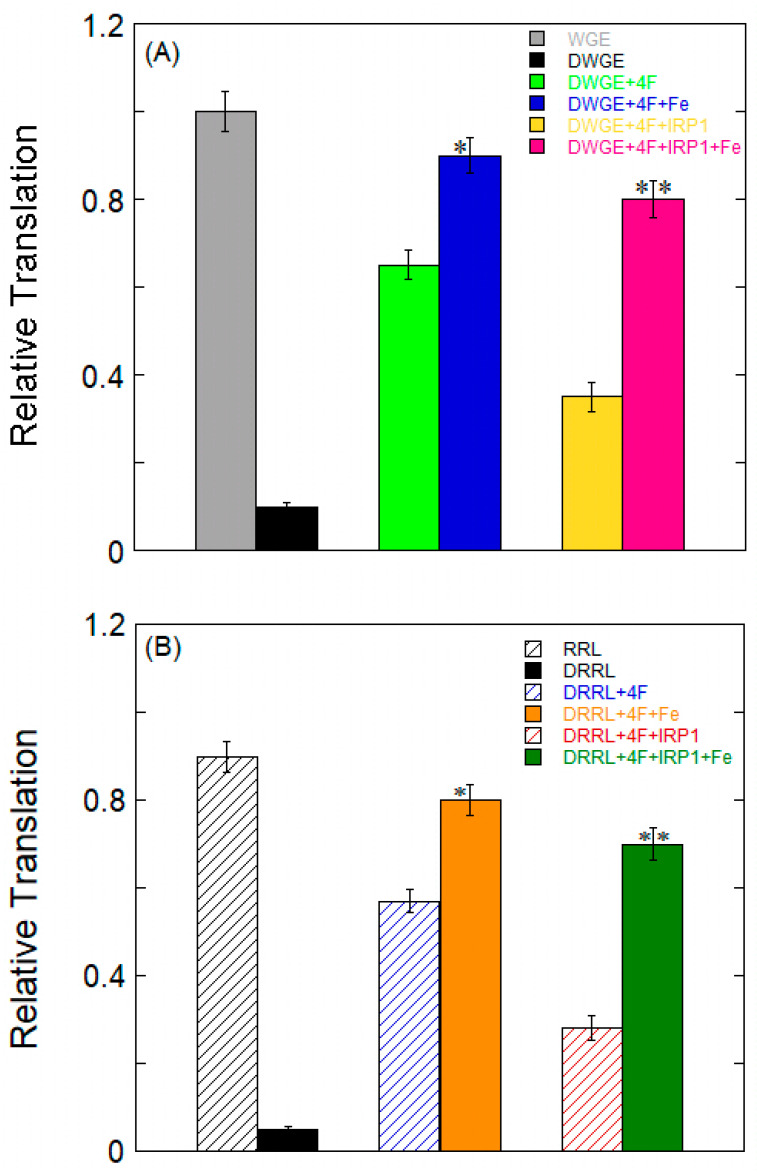
Fe^2+^ enhances translation initiation, which is regulated via α-Syn mRNA. Synuclein mRNA (full-length, poly(A)-tailed, and capped) was translated in (**A**) wheat germ extracts and (**B**) in rabbit reticulocyte lysates. Fe^2+^ changes IRP1 or eIF4F association and increases translation. Syn mRNA was translated in eIF4F-depleted WG extracts and RR lysates that were supplemented with either eIF4F or IRP1 (Syn RNA = 10 nM, eIF4F/IRP1 = 100 nM, Fe^2+^ = 50 μM). * *p* < 0.02, ** *p* < 0.01, as determined by Student’s two-tailed *t*-test. The values plotted are means ± S.E. of n = 3 independent experiments for each translation group.

To examine the possible impact of Fe^2+^ on the translational regulation of protein synthesis in vitro, which is guided by the full-length synuclein mRNA construct, 5′-capped, 3′-polyadenylated mRNA translation was compared in RR lysate and WG lysate. WG lysate and RR lysate were chosen because they have been demonstrated to control ferritin mRNA with translation initiation factors. The preparation of eIF4F-depleted WGE and RRL was carried out as previously described [39,40]. In order to replenish the initiation factor eIF4F during Syn mRNA translation, we used both whole or depleted WG lysate and RR lysate. About 90% less translation occurred when eIF4F was depleted from the WG lysate (Figure 7A) and RR lysate (Figure 7B). By adding exogenous eIF4F, translation was restored to ~65% and 57% for depleted WG lysate and RR lysate, indicating the role of that eIF4F binding to Syn IRE plays in translation. The addition of Fe^2+^ restored the translation to ~90% and 80% for the depleted WG lysate or RR lysate supplemented with eIF4F. The increase in Syn mRNA translation upon the addition of Fe^2+^ to the deficient WG lysate combined with exogenous eIF4F demonstrates the role of Fe^2+^ in improving Syn IRE’s interaction with eIF4F (Figure 6B).

Additionally, the impact of repressor IRP1 on Syn mRNA translation was compared. To find out to what degree translation was repressed, eIF4F-depleted lysate supplemented with eIF4F was used. The level of Syn mRNA translation was approximately 30% that of the level of repression of the syn IRE transcript following the addition of IRP1 in depleted WG lysate or RR lysate with added exogenous eIF4F. These findings suggest that the interaction of IRP1 with Syn IRE specifically prevents translation. Further, by stabilizing the Syn IRE/eIF4F complex and disrupting the Syn IRE/IRP1 complex [29], the addition of Fe^2+^ restored translation for Syn mRNA to ~80% and ~70% for depleted WG lysate or RR lysate with either added eIF4F or IRP1. By causing IRP1 to be released from Syn mRNA and encouraging eIF4F binding to Syn mRNA, Fe^2+^ reversed the effect of IRP1’s suppression of protein synthesis (Figure 7A,B). This result suggests that Fe^2+^ is essential for stimulating the creation of synuclein proteins. The Syn transcripts demonstrating variations in synuclein protein production in RR lysate were reliant on IRP-regulated protein synthesis in wheat germ extract, which lacks IRE recognition proteins (IRPs).

## 3. Discussion

The translation of α-Syn is regulated by a three-dimensionally folded hairpin stem–loop that resembles IREs in the 5′-noncoding region of its mRNA code. Two regulatory proteins (IRP and eIF4F) can be competitively bound by α-Syn IRE, demonstrating the significant impact that untranslated mRNA can have on the expression of genes and the rate of translation. Not only does IRE bind to IRP, a protein synthesis repressor, but it also attaches to the big protein complex (eIF4F), which is known to be an activator of protein synthesis. Different cellular conditions can alter binding to IRE mRNA by phosphorylating the initiation factor protein itself and/or initiation factors binding proteins [41]. Here, we investigated α-Syn IRE’s binding affinity for eIF4F. α-Syn IRE was found to have a high nanomolar affinity interaction with eIF4F. Fe^2+^ further increased the affinity of α-Syn IRE for eIF4F. Adding Fe^2+^ further increased the association between α-Syn IRE and eIF4F by approximately three-fold; however, it decreased the association between α-Syn IRE and IRP1 by nearly three times [31]. Although the associations of APP IRE/eIF4F and Syn IRE/eIF4F are similar in the presence and absence of Fe^2+^, Syn IRE interacts with eIF4F or IRP1 at the same level as APP IRE (nanomolar). Intracellular iron resulted in enhanced intracellular Syn expression in WG lysates by enhancing eIF4F’s binding to Syn IRE. The in vitro activation of α-Syn mRNA protein synthesis after the addition of Fe^2+^ demonstrates the biological relevance of this relationship. Syn IRE interacts with eIF4F with a high association constant, stabilizing complex formation.

The variable IRE/eIF4F stability indicates that the structure of stem–loop IRE is at the 5′-noncoding region of APP, ferritin, and Syn, which distinguishes complex formation. The eIF4F in these complexes is identical. The structural variations in IREs can account for the different ways that IRPs bind iron-responsive elements at the 5′- or 3′-noncoding regions and the wide range of mRNA expression [36]. Compared to the more recently discovered Syn and APP IREs [42,43], ferritin 5′-noncoding IRE is considered an ancient IRE. This suggests that the IRE’s shorter evolutionary fine-tuning time could be the reason for the slower response of Fe^2+^, the reduced stability between Syn IRE and IRP1, and the enhanced stability between Syn IRE and eIF4F. However, the difference in the Fe^2+^ response of these proteins may conceivably reflect each encoded protein’s physiological role. Iron may cause relatively fewer alterations in protein synthesis, for example, because of the detrimental effects of large differences in synuclein protein expression on oxidative metabolism inside cells. However, cells can adjust ferritin synthesis in response to iron fluctuations thanks to ferritin mRNA translation [44]. It is suggested that iron enhances the affinity of Syn IRE for eIF4F and stabilizes the complexes that result in dissociated Syn IRE/IRP1 complexes through labile Fe^2+^ level changes. eIF4F and IRP1 respond to cellular Fe^2+^ iron levels by mediating protein synthesis [28].

Thermodynamic properties have been used to determine the binding forces between various proteins and RNA molecules [37]. The mechanism underlying the interaction between APP and ferritin IRE has been clarified by our previous research on their binding to eIF4F [28] and IRP1 [45]. Syn IRE/eIF4F complex formation is possible at various temperatures, and the binding reaction is more likely to be spontaneous, according to the negative value of ΔG. The binding reaction’s negative enthalpy change indicates that it is exothermic. The negative values for both enthalpy and entropy indicate that the dominating forces in the creation of the Syn IRE/eIF4F complex are van der Waals forces and hydrogen bonding. The enthalpy increased dramatically with the addition of Fe^2+^ (ΔH = −23.6 kJ mol^−1^), indicating that there are more hydrogen bonds when iron is present [45]. It is interesting to note that the addition of Fe^2+^ dramatically alters the value of ΔG for the Syn IRE/eIF4F complex to −51.9 kJ mol^−1^, and the difference in the Gibbs free energy is equal to a shift of roughly 18.7 kJ mol^−1^. There are typically four more hydrogen bonds as a result of this change in binding energy. Our enthalpy shift results are further validated by ΔG contributions to complex stability, which include hydrogen bonding and van der Waals interactions for the association between Syn IRE and eIF4F. The sign and strength of the thermodynamic characteristics demonstrated the contribution of protein–RNA bonding [37,46]. Furthermore, the Syn IRE/eIF4F complex’s stability is primarily determined through these interactions (hydrogen bonding and van der Waals forces), as indicated by the substantial negative ΔG [37,47]. The normal ΔG value difference for the association of the complex represents these interactions [48]. Depending on the ΔG of complex formation, Fe^2+^ affects the stability of the complex through the larger number of bonding forces that cause structural changes for Syn IRE’s association with eIF4F. These results demonstrate that Fe^2+^ affects the Syn IRE/eIF4F complex’s conformational change, which strengthens the hydrogen bonding and may increase binding selectivity.

**Figure 8:**
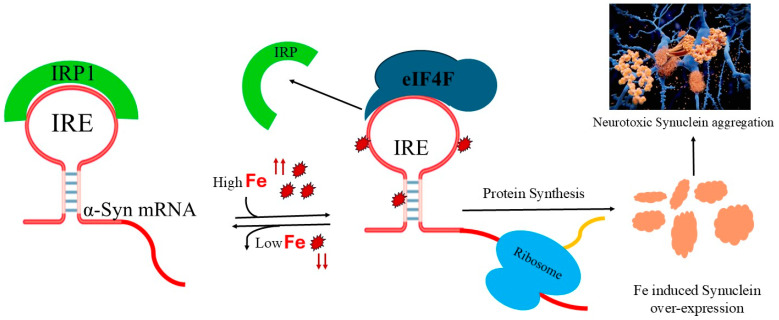
A model for α-Syn IRE mRNA dual-protein (IRP and eIF4F) translational regulation through iron. IRP/Syn IRE binding inhibits Syn IRE/eIFs–ribosome binding. High cellular Fe^2+^ concentrations remove IRP from Syn IRE/IRP binding complex and enhance eIF4F binding to Syn IRE. Syn IRE mRNA/eIF4F recruits ribosomes to begin protein synthesis.

IRP1 and eIF4F effectively compete for Syn IRE mRNA binding. In contrast to IRP1 binding, Fe^2+^ enhanced the binding of Syn IRE/eIF4F. Fe^2+^-dependent Syn mRNA transcript protein synthesis in initiation factor 4F-depleted WG lysates or RR lysates were restored by the exogenous addition of eIF4F. Fe^2+^-stimulated Syn mRNA translation is enhanced (Figure 7), where initiation factor 4F and ribosome binding are facilitated, and the proposed model (Figure 8) shows that Fe^2+^ Syn IRE mRNA decreases the repressor-iron-regulatory protein interaction, whereas it increases the initiation factor 4F (activator) interaction [49]. We demonstrate that Syn may be translated in vitro in an iron-responsive-element-dependent manner in the presence of eIF4F. Even when IRE-dependent translation is inhibited by IRP1, Fe^2+^ stimulates the translation of Syn mRNA. Based on these results, we were able to confirm that the IRE present in the Syn 5′-UTR mRNA controls the translation of synuclein. The activity of Syn IRE was further increased by raising cellular iron levels. Like APP and ferritin IRE-dependent mRNA translation, Fe^2+^ is therefore essential for controlling Syn translation.

It is postulated that Fe^2+^-regulated neurotoxic synuclein protein overexpression reflects the normal Fe^2+^ signal on Syn mRNA translation. PD progresses as a result of the damaging synuclein buildup, which also causes protein aggregation in Lewy bodies. Since both IRE RNAs (Syn and APP) bind with eIF4F and IRP1 with a similarly high affinity, it is plausible that Syn IRE plays a role in brain Fe regulation. Based on the relationship between ferritin and APP IRE’s association with IRP1, the data demonstrated that Fe^2+^ promotes the removal of IRP1, which permits ribosomal binding and initiation factors; consequently, ferritin mRNA translation is increased [28,35,50]. It has been noted that PD patients’ brain tissues contain significantly high levels of iron [51]. In addition to the Fe metabolic proteins (such as ferroportin, mitochondrial aconitase, and ferritin), the IRE family has been extended to include mRNA encoding an α-synuclein, amyloid precursor [31], α-hemoglobin chaperone [52], and cell cycle [53] proteins. These results imply that certain metabolic processes in animal cells are impacted by the regulation of protein synthesis by IRE mRNA. Considering the strong evidence regarding synuclein plaque in the brain cortex and hippocampus regions of Parkinson’s disease, surrounded by higher Fe^2+^ [54] levels, it can be concluded that iron increases synuclein neurotoxicity in the brain areas affected by Parkinson’s disease via neurodegeneration through synuclein aggregation, oxidative stress, and free radical damage.

## 4. Materials and Methods

### 4.1. Preparation of eIF4F, IRP1, and RNA

eIF4F and IRP1 proteins were purchased from Ori-Gene Co. (Rockville, Maryland, USA). Purity of the eIF4F and IRP1 was confirmed by 12% SDS-PAGE (poly acrylamide gel electrophoresis). Human α-Syn IRE RNA oligonucleotide (50-nt) was purchased from Metabion International Co. (Planegg, Germany) Syn IRE was stored at −20 °C until it was needed. Following dissolution, RNA was melted and reannealed in accordance with the prior protocol [35] by heating in 20 mM HEPES (pH 7.2), 0.1 mM EDTA, 1 mM MgCl_2_, and 100 mM KCl for 5 min at 85 °C before gradually cooling to 25 °C [36]. RNA concentration was determined through optical density 260 nm using an optical density value of 40 µg/mL as 1. An A_260/280nm_ ratio of 1.9 was used to measure the synthesized oligonucleotide’s purity. A Bio-Rad (Hercules, CA, USA) protein assay reagent with BSA as a standard was used to measure protein concentrations [55]. Diethylpyrocarbonate-treated water was utilized to prepare all buffers, including RNA.

### 4.2. Fluorescence Spectroscopy Measurements

To analyze α-Syn IRE binding to eIF4F, steady-state fluorescence measurement was conducted under the following experimental conditions: Slit widths of 5 nm for both excitation and emission were used, with a 10 mm pathlength of the cuvette. Fluorescence spectra (λ_em_) were recorded between 300 and 400 nm, whereas excitation (λ_ex_) was measured at 280 nm. α-Syn IRE was used to titrate eIF4F (50 nM) in the concentration range of 0.0 to 500 nM in a titration buffer, 20 mM HEPES, pH 7.4, which contained 1 mM MgCl_2_ and 100 mM KCl. Under the same conditions, appropriate blanks were utilized as controls, and the given fluorescence data were the subtracted spectra. For every sample, thorough degassing was performed. Measurements of fluorescence were made at 298 K unless otherwise noted. For each sample binding investigation, a thermocouple device was used to maintain the temperature (ΔT ± 0.1 °C) of the experimental samples. To assess how the eIF4F fluorescence intensity changed in tandem with the increase in the α-Syn IRE, the following formula was used.

ΔF = (F_0_ − F_f_)/F_0_

where F stands for the observed fluorescence signal of the reaction mixture. The fluorescence signal of eIF4F only (as a control) is represented by F_0_, while after adding α-Syn IRE, the fluorescence change is shown by F_f_. To account for the observed fluorescence signal of the eIF4F/α-Syn IRE complex, a buffer containing the α-Syn IRE intensity was subtracted. The eIF4F/α-Syn IRE binding was calculated using the adjusted fluorescence intensity. The ΔF/ΔF_max_ value was used to calculate the dissociation constant. After adding α-Syn IRE to the sample containing eIF4F, the fluorescence signal variation of eIF4F is represented by ΔF_max_. To estimate ΔF_max_, 1/ΔF versus 1/[α-Syn IRE] was extrapolated [29]. The average value of each titration experiment was provided after each fluorescence titration measurement was performed three times. Non-linear data fitted to the KaleidaGraph program (version 2.1.3; Abelbeck Software) was utilized in order to obtain the binding constant.

### 4.3. RNA Gel Shift Assay

As with the fluorescence experiments, α-Syn IRE RNA was treated with recombinant eukaryotic initiation factor (eIF) 4F protein for RNA electrophoretic mobility shift assays (EMSA). Agarose gels at 1% were used for the electrophoresis mobility shift experiment to resolve the α-Syn IRE RNA/eIF4F complexes. By using ethidium bromide to stain the gel, as previously mentioned, RNA/protein interactions were found [35]. eIF4F protein concentrations ranged from 0.1 to 1.0 μM, while α-Syn IRE RNA concentrations were 0.1 μM.

### 4.4. Fe^2+^ Effect on α-Syn IRE/eIF4F Interaction

We also investigated the effect of Fe^2+^ on α-Syn IRE’s interaction with eIF4F to determine the interaction mechanism. To assess α-Syn IRE’s interaction with eIF4F upon the addition of Fe^2+^ (50 μM), we performed a binding test. α-Syn IRE and eIF4F sample mixtures were supplemented with the same quantity of Fe^2+^ (anaerobic conditions, O_2_). The α-Syn IRE/eIF4F experimental samples were kept in titration buffer at experimental temperature for 15 min. To prevent the ferrous (Fe^2+^) iron from oxidizing to ferric (Fe^3+^) iron, all incubations involving Fe^2+^ were anaerobic [35]. To dissolve FeSO_4_ and stop oxidation, for Fe^2+^, a nitrogen-purged 0.1 M hydrochloric acid solution was diluted to 1mM; for RNA and protein solutions, the acid was further diluted 1:100. As mentioned above for the fluorescence titration measurements, all other experimental conditions were maintained at the same level.

### 4.5. Temperature-Dependent α-Syn IRE/eIF4F Binding

A fluorescence binding study was further carried out to observe the effect of temperature on α-Syn IRE’s interaction with eIF4F both with and without Fe^2+^. The binding of α-Syn IRE with eIF4F was conducted under the same fluorescence titration experimental conditions at five distinct temperatures (283, 288, 293, 298, and 303 K), as previously stated. A temperature-controlled fluorescence instrument was used to maintain the temperature (ΔT ± 0.1 °C) of each sample mixture for all temperature-dependent binding studies. Each sample mixture was stored for 15 min to maintain the required experimental temperature.

### 4.6. Thermodynamic Measurements

As previously mentioned, we employed temperature-dependent binding constant data to assess the thermodynamics of the association between α-Syn IRE and eIF4F [45]. Thermodynamic measures, including the enthalpy change (ΔH), entropy change (ΔS), and free energy change (ΔG), were used to quantify the participation of the interacting forces for the association of α-Syn IRE with eIF4F. To further investigate the stability of the Syn IRE/eIF4F interaction in the presence and absence of Fe^2+^, van’t Hoff plots were employed between binding affinity and temperature. The following isobaric approach was used to measure the enthalpy and entropy contributions for the complex formation:

(1) $$ln\;K_{a\;}=\;-\frac{∆H}{R\;T\;}+\;\frac{∆S}{R}$$

where *K*_a_ depicts the binding affinity (*K*_a_ = 1/*K*_d_). *K*_a_ was measured at five different temperatures (283, 288, 293, 298, and 303 K, respectively). R (1.987 cal mol^−1^ K^−1^) depicts the gas constant, and the observed temperature (T) was measured in K (Kelvin). The intercept and slope of the plot of ln*K*_a_ vs. the inverse of temperature provides the standard enthalpy (–ΔH) and entropy (TΔS). The Gibbs free energy (ΔG) of the association was determined from Equation (2).

(2) $$∆G=∆H-T∆S\;\mathrm{and}\;∆G=-R\;T\;ln\;K_{a}$$

### 4.7. Competitive Binding of IRP and eIF4F for α-Syn IRE

Competitive binding tests were conducted to assess the particular binding location of eIF4F and IRP1 on α-Syn IRE. Following the addition of increasing quantities of IRP1, fluorescence measurements were conducted by tracking the change in intensity of the ^FI^Syn IRE (50 nM). Three distinct levels of eIF4F (0.0, 25, and 50 nM) were added, and samples were incubated for 15 min at 298 K to allow for complex formation. Protein binding to α-Syn IRE was tracked using 5′-fluorescein (FI)-labeled α-Syn IRE. Competitive binding data was evaluated employing a Lineweaver–Burke plot, which compares 1/[IRP1] with 1/F-F_obs_. Competitive binding is shown by lines that intersect at the same place on the y-axis, as opposed to parallel lines that would be interpreted as uncompetitive binding. Non-linear regression analysis was used to fit the data (KaleidaGraph, Abelbeck Software, version 2.1.3).

### 4.8. Protein Synthesis Assays

Translation assays were conducted using Promega wheat germ (WG) lysate and rabbit reticulocyte (RRL) lysates that had been treated with nuclease. Following the manufacturer’s directions, WG lysates and RRL were used to translate the full-length poly(A) tail-capped transcribed synuclein mRNA (IRE luciferase reporter mRNA). In brief, the reaction mixture contained 40 units of RNase ribonuclease, 100 mM KCl, 2 mM MgCl_2_, 25 μL of either WG lysate or RRL, 1 mM amino acids (set of 19 amino acids), and a 10 nM (final concentration) full-length synuclein IRE-luc mRNA template. All protein translation assays involved heating the synuclein RNA to 65 °C for five minutes. Following the addition of 20 mM HEPES, pH 7.2, 50 mM KCl buffer, the translation sample mixture was gradually allowed to cool to 25 °C for half an hour. As previously noted, the ability of eIF4F to stimulate translation was assessed in depleted WG lysate and RRL [46,50]. Depleted WG lysate or RRL were added to the translation mixture together with 100 nM eIF4F in order to examine the eIF4F-dependent translation. To further evaluate the translation of synuclein mRNA in WG lysate or RRL, IRP (100 nM) was added, and the mixture was incubated for half an hour prior to the translation experiment.

In order to facilitate metal binding, samples were incubated at 4 °C for 30 min after Fe^2+^ was added. Anaerobiosis for Fe^2+^ was accomplished in sealed glass vials that had been nitrogen-purged. In order to prepare WG lysate and RRL, depletion of initiation factors was achieved via the previously described methods [39]. After adding 100 μL of the luciferase assay reagent into each translation sample, the amount of protein expression was determined by measuring the absorbance at 495 nm.

### 4.9. Statistical Analysis

Means ± standard deviations (SDs) of the experiments are used to represent values in the text and figures. For each measurement, three different sample experiments were conducted with equal or distinct variation, and the average value of the experimental data is provided. Comparing and assessing statistically significant differences between experimental groups was carried out using two-tailed *t*-tests. The threshold for statistical significance was set at *p* values less than 0.05. The experimental data was statistically processed using the KaleidaGraph software (version 2.1.3; Abelbeck) to create non-linear curve fitting graphs.

## 5. Conclusions

In this study, the molecular interaction between eIF4F and α-Syn IRE was investigated. With a high affinity, α-Syn IRE interacts particularly with eIF4F. Fe^2+^ significantly enhances this interaction. Iron controls both the equilibrium and thermodynamics of the α-Syn IRE/eIF4F interaction, which promotes protein synthesis. The thermodynamic approach provides a direct insight into the energetic and entropic characteristics of the complex formation. With a negative binding energy shift, the reaction is spontaneous. Energy parameters for the Syn IRE/eIF4F association revealed that the main stabilizing forces are hydrogen bonding and van der Waals contacts. Syn IRE has a high binding energy for eIF4F; iron inflow increases the number of hydrogen bonds formed at the RNA–protein contact sites, increasing the complex’s thermostability. Furthermore, in vitro experiments have shown that Syn IRE mRNA transcript at the 5′-noncoding leader can initiate protein synthesis similar to ferritin and APP mRNA translation. These findings support the notion that Syn translation may be just as successful as ferritin and/or APP translation, which is dependent on IRE. As a result, cellular iron levels can control Syn expression at the translation level.

In summary, these results demonstrated that functional IRE encoded by Syn mRNA binds to the translation initiation factor eIF4F with a significantly stronger affinity. This is the first study to investigate the mechanism of the interaction between eIF4F, which is necessary for the production of synuclein in PD, and the therapeutically relevant repressor IRP1 protein. Understanding the mechanism of Syn IRE’s association with initiation factor 4F helps facilitate the comprehension of the dynamics that result in this relationship as well as the details of how Fe^2+^ affects the association between Syn IRE and eIF4F to stabilize this complex and the association of Syn IRE with IRP1 to destabilize the complex. This work sheds light on how iron influx controls the expression of synuclein protein and how Syn IRE binds to eIF4F. Inhibiting the expression of Syn is a successful treatment method for Parkinson’s disease. Crucially, our investigations show that Syn IRE mRNA encodes the structure, offering a way to target structured RNA to prevent synuclein-synthesizing proteins through small molecules. These findings have the potential to significantly improve Parkinson’s disease treatment and pave the way for new clinical medicine directions.

## Figures and Tables

**Table 1:** Interaction of α-Syn IRE with eukaryotic initiation factor 4F with and without Fe^2+^.

Complex			*K*_D_ (nM)		
	10 °C	15 °C	20 °C	25 °C	30 °C
α-Syn IRE∙eIF4F	35.8 ± 1.6	45.6 ± 2.1	69.8 ± 2.8	119.2 ± 6.4	158 ± 8.7
α-Syn IRE∙eIF4F∙Fe^2+^	14.4 ± 0.7	24.6 ± 1.4	38.5 ± 1.9	43.7 ± 2.7	88.2 ± 2.8

**Table 2:** Thermodynamic characteristics for α-synuclein IRE’s association with eIF4F complex stability with or without Fe^2+^ (50 µM).

Complex	Δ*H*	Δ*S*	Δ*G*
	kJ mol^−1^	J mol^−1^ K^−1^	kJ mol^−1^
α-Syn IRE∙4F	−45.6 ± 2.9	−35.7 ± 3.4	−33.2 ± 2.7
α-Syn IRE∙4F-Fe^2+^	−69.2 ± 3.5	−83.9 ± 4.7	−51.9 ± 2.8

## Data Availability

The corresponding author can provide the datasets used and/or analyzed in this study upon reasonable request.
